# An Entertainment-Education Video and Written Messages to Alleviate Loneliness in Germany: Pilot Randomized Controlled Study

**DOI:** 10.2196/43036

**Published:** 2023-06-07

**Authors:** Shuyan Liu, Luisa Wegner, Matthias Haucke, Jennifer Gates, Maya Adam, Till Bärnighausen

**Affiliations:** 1 Department of Psychiatry and Psychotherapy (Campus Charité Mitte) Charité – Universitätsmedizin Berlin Berlin Germany; 2 Columbia University Mailman School of Public Health New York City, NY United States; 3 Icahn School of Medicine New York City, NY United States; 4 Heidelberg Institute of Global Health Heidelberg Germany; 5 Department of Pediatrics Stanford University School of Medicine Stanford, CA United States; 6 Africa Health Research Institute Somkhele and Durban South Africa; 7 Harvard Center for Population and Development Studies Cambridge, MA United States

**Keywords:** entertainment media, perceived social isolation, health communication, digital knowledge

## Abstract

**Background:**

More than half of adults in Germany have felt lonely during the COVID-19 pandemic. Previous studies highlight the importance of boosting positive emotions and social connectedness to combat loneliness. However, interventions targeting these protective psychosocial resources remain largely untested.

**Objective:**

In this study, we aim to test the feasibility of a short animated storytelling video, written messages boosting social connectedness, and a combination of both for alleviating loneliness.

**Methods:**

We enrolled 252 participants who were 18 years or older and spoke fluent German. Participants were recruited from a previous study on loneliness in Germany. We measured the effects of a combination of an animated video and written messages (intervention A), an animated video (intervention B), and written messages (intervention C) on loneliness, self-esteem, self-efficacy, and hope. We compared these with a control arm, which did not receive any intervention. The animated video was developed by Stanford University School of Medicine to reflect experiences of social isolation during the COVID-19 pandemic and convey messages of hope and solidarity. The written messages communicate four findings from recent studies on loneliness in Germany: (1) over a period of 6 months, 66% of respondents in Germany reported feeling lonely (feelings of loneliness are surprisingly common); (2) physical activity can ease feelings of loneliness; (3) focusing on “what really matters” in one’s life can help to ease feelings of loneliness; and (4) turning to friends for companionship and support can ease feelings of loneliness. Participants were randomized 1:1:1:1 to interventions A, B, C, and the control condition, using the randomization feature of the web-based platform “Unipark,” on which our trial takes place. Both the study investigators and analysts were blinded to the trial assignments. The primary outcome, loneliness, was measured using the short-form UCLA Loneliness Scale (ULS-8). Our secondary outcomes included the scores of the Coping with Loneliness Questionnaire, the 10-item Rosenberg Self-Esteem Scale (RSE), the 10-item General Self-Efficacy Scale, and the 12-item Adult Hope Scale (AHS).

**Results:**

We observed no statistically significant effect of the tested interventions on loneliness scores, controlling for the baseline loneliness score before an intervention (all *P* values >.11). However, we observed significantly greater intention to cope with loneliness after exposure to an animated video when compared with the control (*β*=4.14; *t*_248_=1.74; 1-tailed *P*=.04).

**Conclusions:**

Our results provide meaningful evidence for the feasibility of a full-scale study. Our study sheds light on the intention to cope with loneliness and explores the potential for creative digital interventions to enhance this psychological precursor, which is integral to overcoming loneliness.

**Trial Registration:**

German Clinical Trials Register DRKS00027116; https://drks.de/search/en/trial/DRKS00027116

## Introduction

Loneliness raises pressing public health concerns worldwide. The associated adverse effects on mental and physical health can shorten life [1], reduce social functioning (eg, social cohesion, trust, and participation) [2], and incur economic costs (eg, lost workdays and productivity, excess health and social care expenses) [3,4]. Moreover, loneliness appears to exhibit elements of contagion, spreading from person to person within social networks, suggesting that nonlonely individuals who are around lonely individuals tend to grow lonelier over time [5]. During the global COVID-19 pandemic, the public health community has focused special attention on the emerging “loneliness epidemic” [6-8].

More than 1 in 3 people in the United States, including 61% of young adults, faced “serious loneliness” during the COVID-19 pandemic [9]. In Europe, feelings of loneliness among EU residents doubled from 12% in 2016 to 25% in Spring 2020 [10]. In Germany, after the first lockdown in 2020, 66% of respondents reported feeling lonely [11]. Meanwhile, loneliness increased negative mood states including fatigue, anxiety, stress, depression, and unhappiness [12], and was associated with an increase in psychological distress over the first 12 months of the pandemic [13] and an increase in physiological stress during lockdown [14]. In the face of a potential “loneliness epidemic,” innovative interventions and associated research on their efficacy are needed to provide convenient, scalable, and cost-effective methods for tackling loneliness [15,16].

The mechanisms underlying loneliness are not well-understood [1,17]. Loneliness is often associated with lower self-esteem and self-efficacy, negative affect, lower levels of hope, and limited use of coping strategies [1,17-19]. Interventions targeting loneliness often focus on 4 key aspects: changing cognitions (the strategy supported by the most convincing body of evidence), training social skills and participating in psychoeducation, supporting socialization or having a “socially focused supporter,” and engaging with the “wider community” [20,21]. One potential strategy for addressing those 4 aspects and combating loneliness is to embed prosocial and health messages in entertainment media, well-known as entertainment-education (EE) [22,23].

Early EE initiatives drew insights from a wide range of theories [22,24,25], such as social cognitive theory [26], the theory of planned behavior (TPB) [27], the health belief model [28], belief system theory [29], and the elaboration likelihood model [30,31]. The dominant theoretical basis has been Bandura’s [26] social cognitive theory, which suggests that EE encourages observational learning and behavioral modeling. According to Bandura [32], an observer exposed to entertainment media (eg, animated videos) obtains values and standards through imitation of others’ behavior [33]. Thus, what is displayed in entertainment media influences the observers’ understanding of an issue or phenomenon that occurs around them [32,33]. Motivation and perceived self-efficacy for imitating modeled behaviors are proportional to viewers’ wishful identification and perceived similarity with the characters depicted in the EE [25,32]. In addition to Bandura’s [26] social cognitive theory, the TPB suggests that behaviors are influenced by intentions [27]. The change in behavioral intentions reflects 3 types of characters from which observers can learn [34]: positive characters who support a prosocial value, negative characters who reject this value, and transitional characters who change from negative to positive characters over the course of the serial. As such, effective EE is designed to incorporate: (1) appealing storylines, (2) high-quality production, (3) unobtrusive persuasive messages, and (4) high potential for involvement with the characters [35,36]. Despite the rapid growth of EE, studies aimed at measuring its effects have been criticized for lacking rigor [34]. Thus, questions regarding the processes motivating potential behavior change are often left unanswered. This gap suggests a need for controlled experiments using EE content to better understand the theoretical mechanisms through which such content changes attitudes, feelings, and behaviors [25,30]. In this context, EE may change our attitudes toward loneliness (eg, is loneliness harmful to our health?), subjective norms (eg, is loneliness a common experience?), and perceived behavioral control (eg, can I take action against loneliness?), which stimulate the intention to cope with loneliness.

In addition to EE, previous studies have emphasized the importance of using research evidence to promote public health [37]. Indeed, health behavior change interventions are often criticized for lacking a research evidence base [38]. Evidence-based health messages created by researchers can raise awareness and reinforce behavioral change [39].

In our study, we aim to test the feasibility of an animated video, written message, or a combination of both for alleviating loneliness. We hypothesize that combining animated videos with written messages will be a creative, innovative, and feasible approach to tackling loneliness. This combined approach could help individuals alleviate loneliness, improve their intentions to cope with loneliness, increase levels of hope, improve their self-esteem and self-efficacy, and enhance their emotional state.

## Methods

### Participants and Procedure

We conducted a pilot randomized controlled web-based trial in Germany from December 2021 to February 2022 by using the web-based platform Unipark [40]. We enrolled participants who were 18 years or older and spoke fluent German. For recruitment, we contacted 881 participants in a previous study on loneliness in Germany. All participants had consented to be contacted for a future study. They were residents of Germany’s 16 federal states and worked in various fields, such as office administration, health care, education, civil service, sales, agriculture, the arts, sports, and media.

### Ethics Approval

The study was approved by the Ethics Committee of Charité–Universitätsmedizin Berlin (ethics number: EA2/143/20), was performed in accordance with the ethical standards laid down in the 1964 Declaration of Helsinki and was registered on the German Clinical Trials Register [41] on November 24, 2021, with registration number #DRKS00027116. We followed Recommendations for Interventional Trials [42] and Good Clinical Practice guidelines [43]. Participants gave informed consent and received €10 (US $10.90) compensation for their participation.

### Study Intervention

Each participant was exposed to the intervention only once. We measured the effects of a combination of an animated video and written messages (intervention A, 4 minutes long), an animated video (intervention B, 3 minutes long), and written messages (intervention C, 1 minute long), and compared these with a control arm, which did not receive any intervention. The animated video was developed by Stanford University School of Medicine and can be viewed on YouTube [44]. The video did not contain any written or spoken language, and the written messages were written in German. Participants were randomized 1:1:1:1 to interventions A, B, C, and the control condition, using the randomization feature of the web-based Unipark platform on which our trial takes place. Participants in intervention group A watched the video first and then immediately read the message after watching the video. The written message conveyed 4 scientific facts without sound or animation.

The wordless animated video features an emotion-driven story-based portrayal of common experiences living through the COVID-19 pandemic. The narrative begins with people living inside socially isolated bubbles due to COVID-19. Their normal social interactions have been disrupted. The bubbles disappear when the COVID-19 vaccine arrives, allowing children to play together and families to gather. In addition to promoting vaccine confidence, the video is designed to reflect perceptions and experiences of social distancing and social isolation during the COVID-19 pandemic and convey messages of hope and solidarity.

The written messages communicated four findings from recent studies on loneliness in Germany. First, over a period of 6 months, 66% of respondents in Germany reported feeling lonely [11]. Feelings of loneliness are surprisingly common. Second, physical movement can ease feelings of loneliness [45]. Third, focusing on “what really matters” in one’s life could help to ease feelings of loneliness [46]. Fourth, turning to friends for companionship and support can ease feelings of loneliness [46].

### Outcomes

We assessed participants’ sociodemographic characteristics (ie, age, gender, years of education, and annual net income) and vaccination status. Our primary outcome was the sum score of the short-form UCLA Loneliness Scale (ULS-8) [47]. The detailed items of the ULS-8 questionnaire can be found in our previous study [11,13]. ULS-8, composed of 8 items, was rated on a 4-point Likert scale (1-4 points), with a total score ranging from 8 to 32, with higher scores indicating a higher level of loneliness. Our secondary outcomes included the mean score of the intention to cope with loneliness and each sum score of hope, self-esteem, and self-efficacy, which were measured by using 10 items from the Coping with Loneliness Questionnaire [48], the 12-item Adult Hope Scale (AHS) [49], the 10-item Rosenberg Self-Esteem Scale (RSE) [50], and the 10-item General Self-Efficacy Scale (GSES) [51], respectively. The intention to cope with loneliness was rated on a visual analog scale (range from 0 to 100), with higher scores indicating a higher intention to cope with loneliness. AHS was rated on an 8-point Likert scale (1-8 points) with a total score ranging from 12 to 96, with higher scores indicating higher hope. RSE and GSES were evaluated on a 4-point Likert scale (1-4 points) with a total score ranging from 10 to 40 and higher scores indicating higher self-esteem and self-efficacy, respectively. The scales that we used in our study were standard scales that have been validated and shown to be reliable in a German context [46,52-55].

To perform a manipulation check (ie, an attention check), we set up content-based questions geared toward identifying whether participants had paid attention to each intervention to which they were exposed. It consisted of 8 quiz questions in intervention A, 4 questions in intervention B, and 4 questions in intervention C. We excluded inattentive participants, who answered less than 50% of the questions correctly, from the analysis.

To measure emotional responses to the stimuli, we asked participants to rate valence or pleasantness, arousal or excitement [56], and loneliness or coping relevance for both the animated video and written messages by using the horizontal visual analog scale ranging from 0 (not at all) to 100 (very much).

The primary outcome was measured immediately before and after interventions, and the secondary outcome was only measured immediately after interventions. Participants’ emotional responses to the stimuli, sociodemographic characteristics, and vaccination status were assessed at the end of the study.

### Sample Size and Power Considerations

As the purpose of our pilot study was to assess the feasibility of the animated video and written messages to alleviate loneliness, we did not perform a power calculation. Our sample size was at least 50 participants per arm, which would be sufficient to evaluate the study design feasibility [57-60].

### Data Analysis

We performed statistical analyses in R version 4.1.0 (R Foundation for Statistical Computing). To test whether loneliness scores decreased after an intervention, we built up a multiple regression model by using “4 trial arms” as the independent variable and “loneliness scores after an intervention” as the outcome while controlling for loneliness scores before an intervention. As recommended by Senn [61], we included loneliness scores before an intervention as a covariate to adjust the results for potential differences at baseline levels of loneliness. We used dummy coding for 4 trial arms to compare each intervention arm to the control arm as a reference. In addition, we added the covariates, including the intention to cope with loneliness, hope, self-esteem, self-efficacy, age, gender, years of education, and income. We then performed Bonferroni-adjusted significance tests for pairwise comparisons. Secondary outcomes were analyzed by building 4 separate linear models with the independent variable “trial arm” as a factor (the control arm as a contrast reference) and each secondary outcome as a dependent variable. To meet the assumption of having no multicollinearity in multiple regression, we calculated the variance inflation factor values for all independent variables. To compare participants’ emotional responses to the animated video and written messages, we performed independent *t* tests. We compared differences in scores of valence or pleasantness, arousal or excitement, and loneliness or coping relevance between the animated video and written messages, for which 2-tailed *P* values were assumed.

## Results

### Overview

Among 881 participants, a total of 275 participants responded to us, and 23 participants did not want to participate in our study. Our final sample consists of 252 participants (184 females; age range 18-71, mean 33.93, SD 11.84 years) who completed our study. Table 1 shows the demographics of the participants in each group. Of the participants, 91.3% (n=230) were fully vaccinated against COVID-19. Each group had similar vaccination rates, which were aligned with similar attitudes toward COVID-19 vaccines. The mean ULS-8 loneliness scores before and after an intervention were 16.45 and 16.15 (ranging from 8 to 32), respectively.

**Table 1:** Sample characteristics.

Variable			Video and message (n=63)		Video (n=61)		Message (n=64)		Control (n=64)
Age (years), mean (SD)			34.03 (12.32)		33.93 (11.72)		34.44 (12.22)		33.33 (11.34)
**Gender, n (%)**									
	Woman	47 (75)		43 (70)		46 (72)		48 (75)	
	Man	14 (22)		18 (30)		18 (28)		15 (23)	
	Other	2 (3)		0 (0)		0 (0)		1 (2)	
Education years, mean (SD)			15.73 (4.06)		15.52 (3.6)		16.42 (5.58)		15.94 (3.77)
Income category, mean (SD)^a^			3.5 (1.69)		3.6 (1.98)		3.3 (1.61)		3.6 (1.85)
**Vaccinated, n (%)**									
	No	4 (6)		4 (7)		2 (3)		2 (3)	
	Once	1 (2)		4 (7)		0 (0)		2 (3)	
	Twice	21 (33)		18 (30)		21 (33)		18 (28)	
	>Twice	36 (57)		35 (57)		39 (61)		42 (66)	
	Not to say	1 (2)		0 (0)		2 (3)		0 (0)	
ULS-8^b^ loneliness score before intervention, mean (SD)			16.3 (4.8)		17.1 (5.14)		16.5 (5.52)		15.9 (4.61)
ULS-8 loneliness score after intervention, mean (SD)			15.8 (5.04)		17.2 (5.26)		16.0 (5.74)		15.6 (4.85)
Intention to cope with loneliness score after intervention, mean (SD)			60.4 (13.7)		60.1 (12.8)		58.1 (12.1)		56.0 (14.5)
Hope score (AHS^c^) after intervention, mean (SD)			63.2 (8.31)		61.8 (7.47)		64.9 (8.67)		62.6 (6.89)
Self-esteem score (RSE^d^) after intervention, mean (SD)			30.1 (6.68)		29.8 (6.79)		29.9 (6.9)		28.7 (6.29)
Self-efficacy score (GSES^e^) after intervention, mean (SD)			28.6 (5.45)		27.8 (5.29)		29.2 (4.99)		27.7 (5.61)

^a^Annual net income based on 12 income categories: (1) €0-€4999, (2) €5000-€9999, (3) €10,000-€14,999, (4) €15,000-€24,999, (5) €25,000-€49,999, (6) €50,000-€74,999, (7) €75,000-€99,999, (8) €100,000-€124,999, (9) €125,000-€149,999, (10) €150,000-€174,999, (11) €175,000-€200,000, and (12) above €200,000 (a currency exchange rate of €1=US $1.09 is applicable).

^b^ULS-8: UCLA Loneliness Scale.

^c^AHS: Adult Hope Scale.

^d^RSE: Rosenberg Self-Esteem Scale.

^e^GSES: General Self-Efficacy Scale.

### Manipulation Check

In the video and message intervention group, participants answered on average 6.71 (SD 0.55) out of 8 questions correctly. In the video intervention group, participants correctly answered on average 2.93 (SD 0.25) out of 4 questions. In the message intervention group, participants correctly answered on average 3.84 (SD 0.37) out of 4 questions. We excluded participants if they answered less than 50% of the questions correctly; this was not the case in any intervention group. Thus, we conclude that participants paid attention to the video and messages.

To determine the overall effectiveness of an intervention independent of the specific type of intervention, we conducted an independent-sample *t* test. Importantly, these results were not controlled for variation at baseline levels of loneliness within each trial arm and were not corrected for multiple testing. For each intervention, there was a statistically significant reduction in loneliness score after the intervention (mean 16.15, SD 5.24) compared to before the intervention (mean 16.45, SD 5.02; *t*_251_=2.44; *P*=.02). The mean difference in loneliness score was 0.30 (95% CI 0.06-0.54).

**Figure 1:**
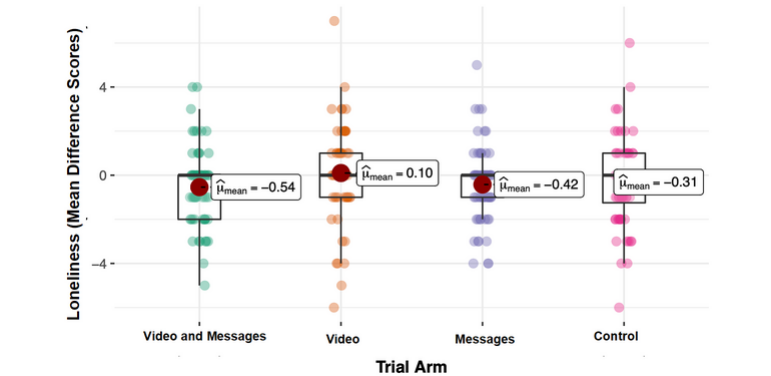
The mean difference between loneliness scores as the primary outcome before and after intervention among the 4 trial arms. The 4 trial arms are a combination of an animated video and written messages, an animated video, and written messages against a control condition. There is no significance in the mean difference in loneliness scores between the 4 trial arms.

To test for specific intervention effects, we conducted a multiple regression analysis. The regression analysis results are displayed in Table 2; all generalized variance inflation factors were <3. In comparison to the control, we did not find a significant effect of the interventions on the loneliness scores after an intervention by adding covariables, including controlling for baseline loneliness scores before an intervention (all *P* values >.11), as shown in Figure 1. However, the baseline loneliness score before an intervention had a significant impact on loneliness scores after an intervention (β=.93; *t*_239_=29.58; *P*<.001). In addition, we found that self-esteem (β=–.065; *t*_239_=–2.103; *P*=.04) and income (β=.229; *t*_239_=2.902; *P*=.004) had a significant effect on loneliness scores after an intervention, reflecting higher self-esteem, and lower income was associated with a lower level of loneliness. As self-esteem and income were significantly associated with loneliness scores after an intervention, we conducted a 2-way ANOVA to compare the effects in each intervention group. We found that there was neither a statistically significant difference in self-esteem (*F*_3,248_=0.594; *P*=.62), nor income between the intervention groups (*F*_3,248_=0.346; *P*=.79).

To calculate effect sizes, we conducted a pairwise comparison of loneliness scores before and after an intervention. The respective effect sizes, *t* values, and *P* values are shown in Table 3. Based on Cohen’s classification of effect sizes (*d*=0.2), medium (*d*=0.5), and large (*d*=0.8) [62], the observed effects are small. Interestingly, “Video and Message” versus “Video” (*d*=–0.319) and “Video” versus “Message” (*d*=0.263) had the highest difference in loneliness scores.

**Figure 2:**
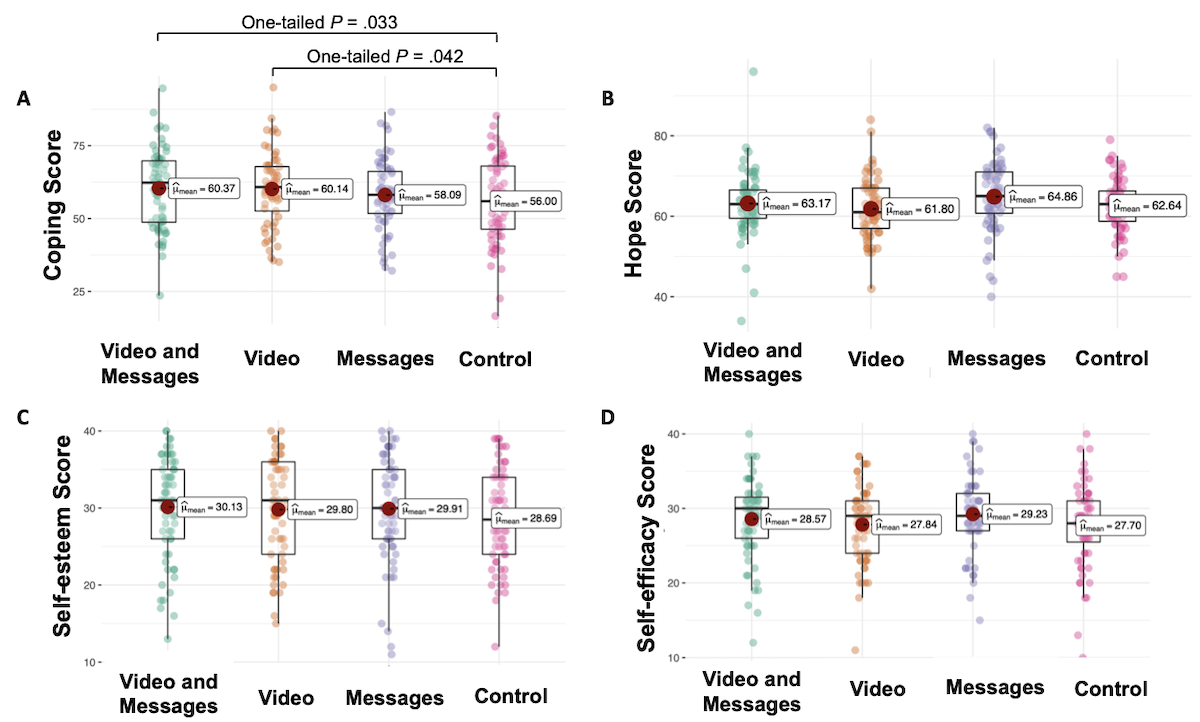
Secondary outcome after an intervention. The differential effects of a combination of animated video and written messages, an animated video, and written messages against a control condition on scores of coping with loneliness (A), hope (B), self-esteem (C), and self-efficacy (D). Significant at *P* (1-tailed test).

For secondary outcomes, we hypothesized that the animated video and written messages would increase intentions to cope with loneliness; therefore, we report 1-tailed *P* values. A linear model showed higher scores of coping with loneliness after exposure to a combination of animated video and written messages (β=4.37; *t*_248_=1.85; 1-tailed *P*=.03) and after watching the animated video (β=4.14; *t*_248_=1.74; 1-tailed *P*=.04) as compared to the control, as shown in Figure 2, although the message did not increase intentions to cope with loneliness (β=2.1; *t*_246_=0.89; 1-tailed *P*=.19).

**Figure 3:**
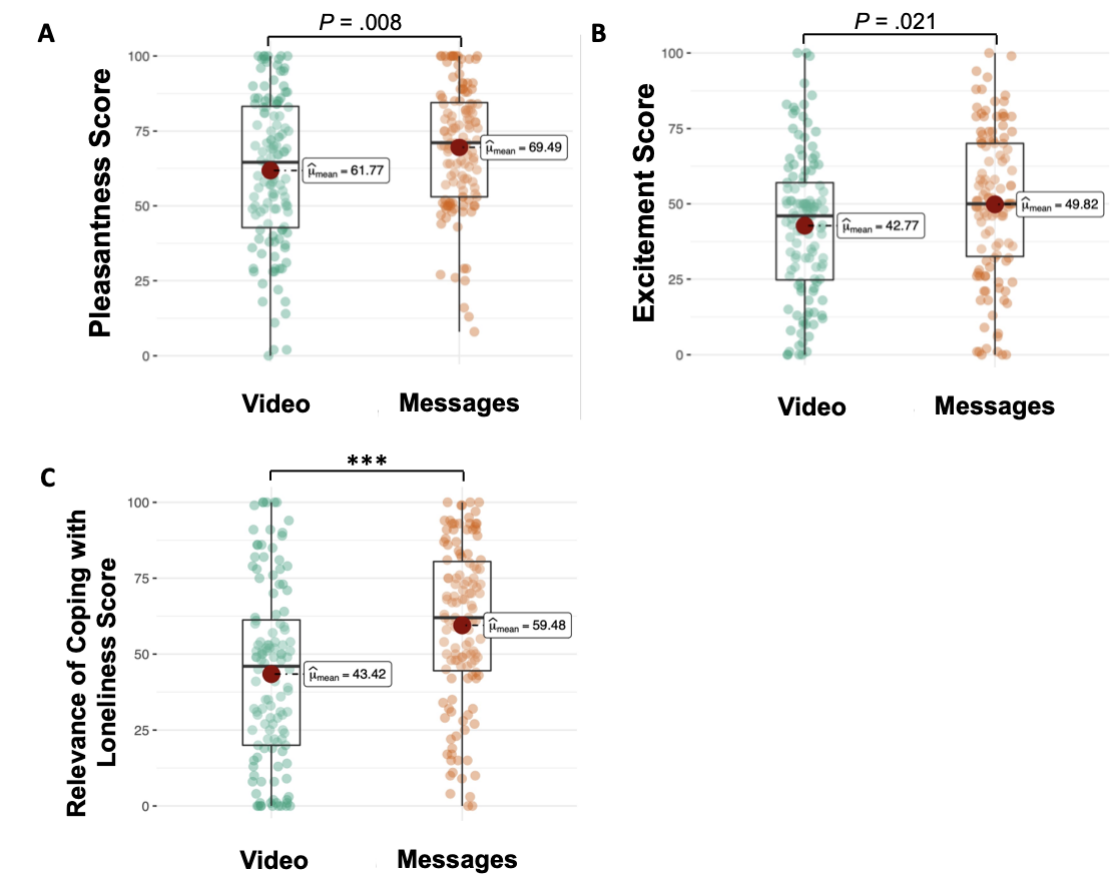
Emotional responses (pleasantness A and excitement B) and the relevance of coping with loneliness (C) to the animated video and written messages. Significant at ***P< .001 (2-tailed test).

Regarding participants’ emotional responses to the animated video and written messages, we found higher valence/pleasantness (*t*_249_=–2.66; *P*=.008), arousal or excitement (*t*_249_=–2.33; *P*=.02), and coping relevance (*t*_249_=–4.56; *P*<.001) to written messages as compared to the animated video, as shown in Figure 3.

**Table 2:** Results of the multiple regression analyses of the loneliness scores after an intervention as an outcome.

Variable			*b*		SE *b*		*T* value		*P* value
Video and message versus control			–0.096		0.342		–0.279		.78
Video versus control			0.558		0.349		1.600		.11
Message versus control			0.076		0.342		0.222		.82
**Covariates**									
	Baseline loneliness scores before intervention	0.926		0.031		29.582		<.001	
	Intention to cope	0.003		0.011		0.275		.78	
	Hope	0.015		0.02		0.750		.45	
	Self-esteem	–0.065		0.031		–2.103		.04	
	Self-efficacy	–0.022		0.038		–0.570		.57	
	Age	0.002		0.012		0.130		.90	
	Gender	0.032		0.257		0.126		.90	
	Years of education	–0.026		0.028		–0.914		.36	
	Income	0.229		0.079		2.902		.004	

**Table 3:** Effect sizes (Cohen d) for the difference in loneliness scores for each trial arm.

Trial arm	*T* test (*df*)	Bonferroni adjusted *P* values	Effect size (Cohen *d*)
Video and message versus control	–0.68 (124)	>.99	–0.120
Video versus control	1.09 (120)	>.99	0.196
Message versus control	–0.33 (124)	>.99	–0.059
Video and message versus video	–1.77 (115)	.40	–0.319
Video and message versus message	–0.38 (125)	>.99	–0.067
Video versus message	1.46 (114)	.80	0.263

## Discussion

In this study, we proposed that combining story-based, animated video content with written messages may be a new and powerful way to convey technical concepts to nonspecialized audiences. However, we found that there was no significant difference in loneliness scores between specific intervention groups after controlling for baseline loneliness scores. Interestingly, we found that overall loneliness scores were lower after exposure to an intervention, regardless of the type of intervention selected. Moreover, we found higher scores on intention to cope with loneliness after exposure to the animated video when compared with the control.

EE videos may be more effective for improving individuals’ intentions to cope with loneliness than reducing feelings of loneliness immediately following exposure. In accordance with the TPB, intentions to change are determined by 3 factors: attitudes, subjective norms, and perceived behavioral control [27]. In this context, there are 3 potential underlying reasons why the intention to deal with loneliness was changed by watching the EE videos. This change of intention may be caused by a change in attitudes toward loneliness, the subjective norm of being lonely, as well as one’s perceived and actual behavioral control over loneliness. Beyond positive and negative characters in EE, transitional characters provide particularly relevant models from which observers can learn [34]. Observers may relate to the uncertainty and doubt transitional characters experience when first considering a new behavior and can observe the characters being rewarded for their adoption of the behavior as the story progresses [63]. A meta-analysis showed that a medium-to-large change in behavioral intentions (*d*=0.66) engenders a small-to-medium change in behavior (*d*=0.36) [64]. Together with previous studies, a digitally animated video can be a science education and communication resource for the general public, but it can also be a tool to improve public health by improving awareness and inspiring people to pursue knowledge [65,66].

In the context of pandemic-related challenges, such as social distancing and increased feelings of loneliness, digital interventions that can reach a broad audience in order to prevent or reduce social isolation and loneliness become even more important [67,68]. The use of short, wordless, and animated video content, designed for rapid dissemination of evidence-based health information, constitutes an innovative approach to supporting global, public responses to crises. [69]. Such interventions have the potential to inspire people to engage in collective action to address social problems [70,71].

Our pilot study played a foundational role in preparation for conducting a full-scale research study. Only 23 out of 275 participants did not want to participate in our study. All participants paid attention to our interventions. They rated the interventions as pleasant, with an average rating above 60 (ranging from 0-100). Effect sizes in our pilot study can be used to determine the sample size for future studies. Given the observed small effect size of the “trial arm,” our power analysis indicated that the estimated sample size for a full-scale study would be 1492 participants. The intention to cope with loneliness was not a primary outcome but a secondary outcome in this study. A full-scale study may include it as a primary outcome and assess participants’ intentions to cope with loneliness both before and after an intervention.

Study limitations include selection bias associated with the recruitment of participants from a previous study on loneliness in Germany. Therefore, any treatment effect of this pilot study may be blunted by this selection bias [72] and the inclusion of a high rate of female participants. The mixed methods intervention design may have enriched the intervention evaluations. Moreover, the mean ULS-8 loneliness scores before and after an intervention were not very high, with 16.45 and 16.15 (ranging from 8 to 32), respectively. This may reflect that our participants had low levels of loneliness, which might have led to a “floor effect” [73]. Thus, it remains possible that our interventions might have high-level effects among participants who reported high levels of loneliness.

To conclude, we found that animated videos increased participants’ intentions to cope with loneliness but did not decrease loneliness scores. Our novel approach lays the foundation for assessing to what extent the components of digital interventions to reduce loneliness are “transferable” to other settings (eg, in different countries) and whether they are likely to result in the same or similar impacts. An effective early intervention to tackle loneliness and social isolation amid COVID-19 and beyond will be key to both better health outcomes and lower health and social care costs in the long term.

## Appendix

Multimedia Appendix 1 CONSORT-eHEALTH checklist (V 1.6.1).

## Abbreviations

AHS: Adult Hope Scale
EE: entertainment-education
GSES: General Self-Efficacy Scale
RSE: Rosenberg Self-Esteem Scale
TPB: theory of planned behavior
ULS-8: UCLA Loneliness Scale

## Data Availability

Data are available with restrictions due to confidentiality in line with the Berlin Data Protection Act (Berliner Datenschutzgesetz, BlnDSG). Interested individuals can contact stresshealth@charite.de.
